# Adipocyte-rich microenvironment promotes TNBC progression through SIRT6-associated ACSL5 dysregulation and lipid storage-associated phenotypes

**DOI:** 10.1186/s13046-026-03769-5

**Published:** 2026-07-01

**Authors:** Yingnan Li, Xinyi Sun, Jing Peng, Teng Ma, Changgen Liu, Haibo Wang

**Affiliations:** Breast Disease Center, Affiliated Hospital of Qingdao University, No. 16 Jiangsu Road, Shinan District, Qingdao, Shandong Province 266000 China

**Keywords:** ACSL5, Lipid storage-associated phenotypic changes, TNBC, Breast adipocyte microenvironment, Lung metastasis

## Abstract

**Background:**

Triple-negative breast cancer (TNBC) is highly aggressive and has strong metastatic potential, and effective targeted therapies remain limited. There is growing evidence that the adipocyte microenvironment plays an important role in tumor progression, but its specific mechanism remains to be studied. As a key enzyme for the activation of long-chain fatty acids, the role and regulatory mechanism of ACSL5 in TNBC have not been clarified.

**Methods:**

ACSL5 expression and clinical relevance were assessed using public datasets, clinical specimens, and TNBC cell models. The effect of ACSL5 knockdown and overexpression on the malignant phenotype of TNBC cells were evaluated. An adipocyte-TNBC co-culture system, PA/OA treatment, SIRT6 manipulation, co-immunoprecipitation, pan-acetylation assays, and ACSL5 K212/213 mutant analysis were used to investigate the SIRT6-ACSL5 regulatory mechanism. Lipid-associated phenotypes were examined by Oil Red O staining, TG measurement, FASN/CPT1A detection, and Seahorse analysis. In vivo validation was performed using an orthotopic mammary fat pad model in female NOD/SCID mice.

**Results:**

ACSL5 expression was reduced in TNBC tissues and cell lines, and lower ACSL5 expression was associated with aggressive disease features. Functionally, ACSL5 knockdown promoted TNBC cell viability, colony formation, migration, invasion, and EMT-associated changes, whereas ACSL5 overexpression exerted opposite effects. Adipocyte co-culture and PA exposure induced SIRT6 upregulation and ACSL5 downregulation, while SIRT6 knockdown partially restored ACSL5 expression. Mechanistically, SIRT6 interacted with ACSL5 and regulated ACSL5 pan-acetylation. Mutagenesis of a candidate K212/213-containing lysine motif suggested that this region may contribute to ACSL5 acetylation-associated functional regulation. Adipocyte co-culture and SIRT6 overexpression increased lipid droplet accumulation, intracellular TG storage, FASN expression, and EMT-associated changes, while reducing CPT1A expression. ACSL5 restoration partially reversed these lipid storage-associated malignant phenotypes. In an orthotopic mammary fat pad model using female NOD/SCID mice, ACSL5 overexpression suppressed tumor growth, lung metastatic involvement, Ki67 expression, tumor TG accumulation, and lipid metabolism-related marker changes, whereas ACSL5 knockdown produced the opposite effects.

**Conclusion:**

These findings suggest that adipocyte-derived fatty-acid cues may promote TNBC progression partly through SIRT6-associated ACSL5 dysregulation and lipid storage-associated phenotypic changes, highlighting ACSL5 as a potential lipid-related regulatory node in adipocyte-driven TNBC progression.

**Supplementary Information:**

The online version contains supplementary material available at https://doi.org/10.1186/s13046-026-03769-5.

## Background

Triple-negative breast cancer (TNBC) is the most aggressive and heterogeneous subtype of breast cancer, accounting for about 15%-20% of breast cancer cases [1, 2]. Although traditional treatment regimens can temporarily inhibit the development of TNBC, the high metastasis rate of TNBC (especially lung metastasis), poor prognosis, and easy recurrence still cause the survival rate of patients to be less than 70% within 5 years [1, 3, 4]. TNBC lacks targeted therapy due to the deletion of estrogen receptor (ER), progesterone receptor (PR) and human epidermal growth factor receptor 2 (HER2), and the treatment options are limited, which exacerbates the generation of drug resistance and the emergence of tumor heterogeneity [4–6], and there is currently no effective targeted treatment strategy [3]. In addition, TNBC is more dependent on its supportive role in the tumor microenvironment (TME) due to the deficiency of ER, PR, and HER2 [7, 8]. Studies have shown that TME plays an important driving role in TNBC, especially adipocytes in TME [7, 9, 10].

The mammary gland is composed of a large amount of adipose tissue, and adipocytes become active signaling and nutrient sources in the TME [8, 11], and their secretion and release of fatty acids and glycerol and other molecules are used by cancer cells, thus reshaping the metabolic mode of breast cancer cells, further promoting the epithelial-mesenchymal transition (EMT), and improving the ability of cell invasion and migration [9, 12]. However, there are few studies on how adipocytes regulate lipid metabolism in the tumor microenvironment, thereby promoting TNBC metastasis and invasion, and there is a lack of research and elucidation on key nodes.

Acyl-CoA synthetase long chain family member 5 (ACSL5) is a key rate-limiting enzyme in fatty acid (FA) metabolism [11], and its main function is to catalyze the synthesis of acyl-CoA of long-chain fatty acids, participate in fatty acid β-oxidation (FAO), maintain lipid homeostasis, and provide conditions for mitochondrial oxidation and energy supply [13–15]. As a member of the long-chain acyl-CoA synthetase (ACSLs) family, its function on tumors is slightly lacking compared to ACSL4, which has been widely studied [1, 16]. Some studies have shown that ACSL5 is significantly underexpressed in tumors and is closely related to poor prognosis [17, 18]. ACSL5 can promote FAO and reduce lipid accumulation, thereby inhibiting the progression of bladder cancer, colon cancer, liver cancer, etc [17, 19–21]. However, its role in triple-negative breast cancer is still scarce, and only a small number of reports of low expression of ACSL5 in breast cancer tissues are associated with poor prognosis [15, 18, 22]. Therefore, whether ACSL5 is involved in the metabolic remodeling of adipocytes and tumor microenvironment in TNBC, which is highly dependent on lipid metabolism, needs to be further elucidated and systematically studied. Given that the breast microenvironment is rich in lipids and ACSL5 plays a central role in long-chain fatty acid activation, we hypothesize that ACSL5 may represent a tumor cell metabolism node linking adipocyte-derived lipid signaling to malignant progression in TNBC. Therefore, we do not interpret ACSL5 as a lineage-specific marker, but rather as a preferred candidate marker for subsequent tumor cell function-related studies under adipocyte conditions.

As an important protein post-translational modification method [23, 24], protein acetylation plays a crucial role in a wide range of biological processes, such as chromatin remodeling, metabolic enzyme activity, signal transduction, subcellular localization and stability [25, 26]. According to in-depth research in recent years, protein acetylation is closely related to the development of a variety of cancers [27], and has become one of the hot spots for targeted cancer therapy [28, 29]. 

SIRT6 is an NAD⁺-dependent deacetylase involved in chromatin regulation, metabolic homeostasis, DNA damage responses, and cancer progression [30]. It is worth noting that the function of SIRT6 is highly dependent on the metabolic state of cells and the tumor microenvironment [24], so it plays a role in promoting or inhibiting the development of different types of tumors [23, 27, 30]. In breast cancer, elevated SIRT6 expression has been shown to be significantly associated with poor prognosis and tumor metastasis [30]. Other studies have reported that SIRT6 can interact with lipid metabolizing enzymes to exert the role of deacetylation modification [23]. Previous studies have shown that SIRT6 can activate its FAO activity by deacetylating ACSL5 [24]. However, their regulatory significance in tumor and metabolic stress conditions is unclear, especially in the tumor adipocyte microenvironment, whether ACSL5 affects the balance of lipid metabolism due to the protein modification of SIRT6, which in turn causes TNBC invasion and migration. Considering that ACSL5 is a lipid metabolism-related enzyme and that adipocyte-derived stimulation may alter the metabolic state of TNBC cells, we hypothesized that SIRT6 may serve as a candidate upstream regulator linking adipocyte-derived fatty-acid cues to ACSL5 dysregulation. Therefore, this study focused on whether SIRT6 interacts with ACSL5 and regulates ACSL5 pan-acetylation, rather than assuming a direct transcriptional mechanism or a specific acetylation site.

Based on these considerations, the present study aimed to clarify the role of ACSL5 in TNBC progression and to determine whether the adipocyte-rich mammary microenvironment contributes to ACSL5 dysregulation through a SIRT6-associated mechanism. We first evaluated ACSL5 expression and clinical relevance using public datasets, clinical specimens, and TNBC cell models.

We then investigated the tumor-cell-intrinsic function of ACSL5 and examined whether adipocyte co-culture and fatty-acid exposure regulate the SIRT6-ACSL5 axis. Mechanistically, we assessed the interaction between SIRT6 and ACSL5 and the regulation of ACSL5 pan-acetylation. Finally, we evaluated the in vivo relevance of ACSL5 using an orthotopic mammary fat pad model in female NOD/SCID mice. This study provides a mechanistic framework suggesting that adipocyte-derived fatty-acid cues may promote TNBC progression partly through SIRT6-associated ACSL5 dysregulation and lipid storage-associated phenotypic changes.

## Methods

### Public databases and bioinformatics analysis

The expression of ACSL5 in pan-cancer and breast cancer was analyzed through open databases.

Transcriptome data of 33 cancers were obtained using TCGA database, and expression difference analysis and visualization were carried out by R software. The mRNA expression levels of ACSL5 in breast cancer tissues in the TCGA-BRCA cohort were compared with those in normal breast tissues by GEPIA platform. The prognostic value of ACSL5 in breast cancer patients was performed by Kaplan-Meier survival assay using the bc-GenExMiner platform. ROC curves were used to evaluate the diagnostic efficacy of ACSL5 for breast cancer.

TNBC-related differential expression analysis was performed using the dataset published in the GEO database (such as GSE113865), and R software was used for standardized processing and differential analysis. Single-cell RNA sequencing data were obtained from the GEO database and were used for cell clustering, annotation, and ACSL5 expression visualization using the Seurat package. Pathway enrichment analysis was performed using ssGSEA in the TCGA-BRCA cohort, and samples were divided into ACSL5-high and ACSL5-low groups according to the median ACSL5 expression level.

### Cell lines and cell cultures

Human triple-negative breast cancer (TNBC) cell lines MDA-MB-231, MDA-MB-468 and BT-549, non-TNBC breast cancer cell lines MCF-7, BT-474 and SK-BR-3, and normal breast epithelial cell line MCF-10 A were all purchased from the Wuhan Pricella Biotechnology Co., Ltd. MDA-MB-231 and BT-549 cells were cultured in 10% fetal bovine serum (FBS; Gibco, USA) and 1% penicillin-streptomycin (P/S; Gibco, USA) Dulbecco’s Modified Eagle’s Medium (DMEM; Gibco, USA). MCF-7, BT-474, SK-BR-3, and MDA-MB-468 cells were cultured in RPMI-1640 Medium (Gibco, USA) containing 10% FBS and 1% P/S. MCF-10 A cells were cultured in complete medium prepared by DMEM/F12 (Gibco, USA). The cells were cultured in a humid incubator at 37 °C and 5% CO_2_.

### Establish a breast adipocyte-tumor microenvironment co-culture system

Sections were prepared from tumor or adjacent tissue of patients with TNBC for subsequent immunohistochemical staining; mature human adipocytes were also isolated from healthy breast adipose tissue. This study strictly adhered to the principle of informed consent to protect the rights and interests of the subjects. After the obtained breast adipose tissue was digested by collagenase, preadipocytes were collected by centrifugation and induced for 14 days using adipogenic differentiation induction medium (Gibco, USA) until significant lipid droplets appeared in the cells.

Differentiated mature adipocytes (Adipocytes) were used for co-culture experiments. Co-culture experiments were performed in a 6-well Transwell (Corning, USA) system. MDA-MB-231 was seeded in the lower chamber at 5 × 10^4^ cell/well density supplemented with DMEM medium containing 10% FBS, and Adipocyte was inoculated in the upper chamber at 2.5 × 10^5^ cell/well density with a cell ratio of 1:5 for a total of 48 h. The control group (Ctrl) was cultured separately for MDA-MB-231 cells.

### Plasmid construction and cell transfection

The expression and interfering plasmids used in the functional study of ACSL5 and SIRT6 in this study were designed according to human gene sequences and synthesized and constructed by Suzhou GenePharma Co., Ltd. The expression vectors (OE-ACSL5 and OE-SIRT6) carrying the full-length coding sequences of the corresponding genes were used in the ACSL5 and SIRT6 overexpression experiments, and the empty vector was used as a negative control (Vector). In the gene knockdown experiment, two independent interference sequences were designed for human ACSL5 and SIRT6 to reduce the potential off-target effect, among which the ACSL5 interference sequences were ACSL5-1 (5′-CACCGATCAACGCCGGGGTCGGAAG-3′ / 5′-GTGGCTAGTTGCTTCCCCCCAGCCTTC-3′) and ACSL5-2 (5′- CACCGATGCAGATCAACGCCGGGGT-3′ / 5′-GTGGCTACGTCTAGTTGCGGCCCCCCA-3′), SIRT6 interference sequences are SIRT6-1 (5′-AAGCTGGAGCCCAAGGAGGAAA-3′/5′-TTCGACCTCGGGTTCCTCCTT-3′) and SIRT6-2 (5′- AAGAATGTGCCAAAGTGTAAGA-3′/5′-TTCTTACACGGTTCACATTCT-3′), randomized sequence as negative controls (shNC or siNC).

To explore the potential involvement of the candidate K212/213-containing lysine motif in ACSL5 acetylation-associated regulation, ACSL5-K212/213R and ACSL5-K212/213Q mutants were constructed. In these mutants, the two adjacent lysine residues within the wild-type sequence NEAKTPLKKFLLKLAV were replaced with arginine residues or glutamine residues, generating NEAKTPLRRFLLKLAV and NEAKTPLQQFLLKLAV, respectively. All constructs were generated using the same vector backbone and confirmed by sequencing.

MDA-MB-231 and BT-549 cells were used for in vitro functional and mechanistic experiments.

Cells are inoculated in 6-well plates or corresponding culture vessels the day before transfection to achieve approximately 60%-70% cell confluency at the time of transfection. Transfection was performed with Lipofectamine 3000 Transfection Reagent (Invitrogen, USA) and operated according to the manufacturer’s instructions. After 6 h of transfection, it was replaced with a complete medium containing 10% fetal bovine serum to continue culture. Cells were collected 24–48 h after transfection for RT-qPCR, Western blot, immunoprecipitation, immunofluorescence, and cell function and metabolism.

### RNA extraction and reverse transcription quantitative PCR (RT-qPCR)

Total RNA was extracted using TRIzol reagent (Invitrogen, USA) by chloroform method. RNA concentration and purity were detected by a NanoDrop One spectrophotometer (Thermo Fisher Scientific, USA). cDNA synthesis was done using a reverse transcription kit (Takara, Japan).

Real-time PCR was amplified using SYBR Green fluorescent dye (Takara, Japan) on a QuantStudio^TM^5 Real-Time PCR System (Thermo Fisher Scientific, USA). The mRNA expression levels of ACSL5 and SIRT6 were normalized using ACTB as an internal reference, and the relative expression levels were calculated by the 2^−ΔΔCt^ method. The primer sequences used were as follows: ACSL5 forward primer was 5′-CTCAACCCGTCTTACCTCTT-3′, and reverse primer was 5′-GCAGCAACTTGTTAGGTCATTG-3′; the forward primer of SIRT6 was 5′-CCCACGGAGTCTCTGGACCAT-3′, and the reverse primer was 5′-CTCTGCCAGTTTGTCCCTG-3′; the forward primer of ACTB was 5′- CATGTACGTTGCTATCCAGGC-3′, and the reverse primer was 5′- CTCCTTAATGTCACGCACGAT-3′. The primers are synthesized by BGI Genomics Co., Ltd. All RT-qPCR reactions were set up with triple wells and at least three independent replicates.

### Western blot

Cell or tissue samples were lysed on ice using RIPA lysate (Beyotime, China) to obtain total protein.

After the lysate is centrifuged to remove insoluble impurities, the supernatant is taken for subsequent testing. The protein concentration was determined by BCA method, and the samples in each group were loaded according to the principle of equal amount of protein. The protein samples were separated by SDS-PAGE electrophoresis and transferred to PVDF membranes. The membrane was incubated at room temperature in 5% BSA, including ACSL5 (1:1000), SIRT6 (1:1000), E-cadherin (1:1000), N-cadherin (1:1000), Snail (1:1000), Vimentin (1:1000), FASN (1:1000), CPT1A (1:1000), and acetylated lysine (1:1000) antibodies (Abcam, UK) and others. The HRP-labeled secondary antibody (Beyotime, China) was then incubated, and the protein signal was detected by chemiluminescence development. β-actin (1:5000) was used as an internal control protein for normalization analysis. The grayscale value of the bands was quantitatively analyzed by ImageJ software. All experiments were repeated at least three times independently.

### Co-Immunoprecipitation (Co-IP) and acetylation assays

Co-IP assays were used to detect the interaction between ACSL5 and SIRT6 and the changes in ACSL5 acetylation levels. After the cells were collected under the corresponding treatment conditions, they were lysed on ice with mild lysis buffer to preserve protein interactions, and the lysate was centrifuged to remove cell debris and the supernatant was taken for subsequent experiments. To reduce non-specific binding, the supernatant was first pre-cleared with Protein A/G magnetic beads. The pretreated lysate was added with anti-ACSL5 or anti-SIRT6 primary antibody, and incubated overnight at 4 °C, followed by the addition of Protein A/G magnetic beads to continue incubation to capture the immune complex. After the magnetic beads were washed several times with lysis buffer, SDS protein loading buffer was added to boil to release the binding protein.

The samples were transferred to the membrane by SDS-PAGE electrophoresis and detected by Western blot. ACSL5 acetylation levels were detected by using anti-acetylated lysine antibodies after immunoprecipitation. IgG as a negative control to assess non-specific binding.

### Immunofluorescence (IF)

Cells were cultured on sterile coverslips to the appropriate density, fixed with 4% paraformaldehyde, and then permeabilized with 0.1% Triton X-100. After PBS washing, cells are blocked at room temperature in a blocking solution containing 5% bovine serum albumin to reduce non-specific binding. After blocking, the cells were incubated at 4 °C for the corresponding primary antibody, including ACSL5 or SIRT6 antibodies. After washing by PBS the next day, the corresponding fluorescently labeled secondary antibody was added and incubated at room temperature under dark conditions. Nuclei were counterstained using DAPI (Beyotime, China). After staining, the coverslips were sealed and observed and acquired using a fluorescence microscope (KEYENCE, Japan).

### CCK-8 cell viability assay

Cell viability was detected by Cell Counting Kit-8 (Beyotime, China). Transfected or treated MDA-MB-231 and BT-549 cells were seeded at the same density in 96-well plates and cultured at 37 ℃ at 5% CO₂. CCK-8 working solution was added to each well at a set time point, and after continued incubation, absorbance values at 450 nm were measured using a Varioskan™ LUX microplate reader (Thermo Fisher Scientific, USA).

### Colony formation assay

The treated cells were seeded in 6-well plates at low density and incubated continuously for about 14 days under complete medium conditions, during which the medium was changed periodically.

After the formation of a macroscopic clone, the cells were fixed with 4% paraformaldehyde and stained with crystal violet (Beyotime, China). The number of clones was counted under a microscope (ZEISS, Germany) to evaluate the effect of different treatments on the ability of cell clone formation.

### Cell cycle analysis

The treated cells were collected and washed with pre-cooled PBS and fixed overnight at 4 °C with 70% ethanol. The fixed cells were washed by PBS and incubated in a dark shade with propidium iodide (PI) staining solution containing RNase A (Beyotime, China). After staining, the proportions of cells in G0/G1, S and G2/M phases were analyzed using a BD Accuri C6 flow cytometer (BD, USA).

### Cell migration and invasion assays

Cell migration and invasion capabilities were detected and analyzed by Transwell chamber experiments. In the migration experiment, the treated cells were resuspended in serum-free medium and inoculated in the upper chamber, and serum-containing medium was added to the lower chamber as chemotaxis. In the invasion experiment, the upper chamber is pre-laid with Matrigel (Corning, USA) to simulate the extracellular matrix barrier, and the rest of the steps are the same as in the migration experiment. After a certain period of culture, the untransembranous cells are gently removed, and the cells transmembrane to the lower chamber are fixed, stained, and counted under a microscope to assess cell migration and invasion capacity.

### Oil Red O staining

Cells were washed with PBS at appropriate time points, fixed with 4% paraformaldehyde and pretreated with 60% isopropanol. Then add oil red O working solution (Solarbio, China) for staining, remove excess dyeing solution and wash thoroughly. The staining results were observed and imaged under a microscope to assess the distribution of lipid droplets in cells under different treatment conditions. For semi-quantification, Oil Red O staining was eluted with isopropanol, and absorbance was measured at 510 nm using a microplate reader.

### Triglyceride assay

Intracellular and tumor tissue triglyceride levels were measured using the Triglyceride (TG) Content Assay Kit (Solarbio, China) according to the protocol. Briefly, cultured MDA-MB-231 cells were washed twice with cold PBS, harvested, and lysed in TG assay lysis buffer. Orthotopic tumor tissues were weighed and homogenized in cold lysis buffer on ice. Cell lysates and tissue homogenates were centrifuged at 12,000 × g for 10 min at 4 °C, and the supernatants were collected for TG measurement. The enzymatic reaction mixture was prepared according to the kit instructions, and absorbance was measured using a microplate reader at the indicated wavelength. TG concentrations were calculated from a standard curve. Total protein concentration was determined using a BCA protein assay kit, and TG content was normalized to total protein concentration and expressed as µg/mg protein. Each sample was assayed in triplicate.

### Cellular energy metabolism analysis

The state of cellular energy metabolism was measured by the Seahorse XF Analytical System (Agilent, USA). Cells are seeded in special microplates, and pretreatment and media replacement are carried out according to instrument requirements before testing. Extracellular acidification rate (ECAR) and oxygen consumption rate (OCR) were monitored in real time during the detection to reflect cellular glycolytic activity and mitochondrial oxidative phosphorylation levels, respectively.

All experiments were performed under the same cell density and uniform detection parameters to ensure comparability of results between different treatment groups.

### NEFA assay

Extracellular free fatty acid levels were assessed by non-esterified fatty acid (NEFA) assay, cell culture supernatant was collected, and after centrifugation to remove cell debris, the supernatant was taken for subsequent testing. The NEFA content was determined by the kit method (Elabscience, China), the reaction steps were completed according to the instructions, and the corresponding absorbance values were determined on the microplate reader.

### Immunohistochemistry (IHC)

Paraffin-embedded tissue sections were deparaffinized, rehydrated, and subjected to antigen retrieval. After blocking endogenous peroxidase activity and nonspecific binding, sections were incubated with primary antibodies against ACSL5 or Ki67 at 4℃ overnight. The sections were then incubated with HRP-conjugated secondary antibody, developed with DAB substrate, counterstained with hematoxylin, dehydrated, and mounted. Representative images were acquired under a microscope. Staining intensity and percentage of positive cells were independently assessed by two pathologists who were blinded to clinicopathological information. The H-score for each sample was calculated by multiplying the staining intensity (0–3) by the percentage of positive cells, and the final score used for analysis was the average of the two observers.

### Establishment of subcutaneous xenograft tumor

Female BALB/c nude mice (body weight 18–22 g, 6–8 weeks of age) were used for subcutaneous xenograft tumor models. MDA-MB-231 cells stably transfected with the corresponding plasmid or control vector were prepared as single-cell suspensions and injected subcutaneously into the back of mice. After inoculation, the status of mice is regularly monitored and tumor volume is measured to assess tumor growth dynamics. The mice were sacrificed and the tumor tissue was stripped at the end of the experiment, and the tumor weight of the endpoint was recorded for subsequent histological and molecular detection analysis.

All experimental animals were purchased from Beijing Vital River Laboratory Animal Technology Co., Ltd., randomly grouped and kept in standard breeding cages. Keep the temperature and humidity in the animal room within the appropriate range, and change the bedding regularly to keep the environment clean. Continue to observe the general condition of the animals throughout the experiment, and if there is obvious discomfort or the humane endpoint is met, it will be treated according to the specifications. All experimental procedures were performed in accordance with the guidelines approved by the Animal Ethics Committee of the Affiliated Hospital of Qingdao University.

### Orthotopic mammary fat pad xenograft model

To establish the orthotopic mammary fat pad xenograft model, female NOD/SCID mice aged 6–8 weeks were used (*n* = 5 per group). MDA-MB-231-luc cells stably expressing shACSL5, OE-ACSL5, or the corresponding control vectors were harvested during the logarithmic growth phase and resuspended in sterile PBS. A total of 1 × 10⁶ cells in 50 µL suspension were injected into the left fourth mammary fat pad of each mouse. Tumor growth was monitored regularly using caliper measurements, and tumor volume was calculated as: volume = length × width^2^/2. For bioluminescence imaging, mice were intraperitoneally injected with D-luciferin at 150 mg/kg and imaged using the IVIS Lumina Series III system under identical acquisition settings. At the experimental endpoint, mice were euthanized, and orthotopic tumors and lung tissues were collected.

### Statistical analysis

Data were analyzed with GraphPad Prism 8.0 and the results were expressed as mean ± standard deviation (mean ± SD). The two-sided Student’s t-test was used for comparison between the two groups, and one-way analysis of variance (one-way ANOVA) was used for comparison between multiple groups. Kaplan-Meier curve log-rank test for survival. *P* < 0.05 is considered significant.

All *in vitro* experiments were performed three independent replicates.

## Results

### ACSL5 was significantly underexpressed in TNBC and was associated with poor prognosis and metastasis

**Fig. 1:**
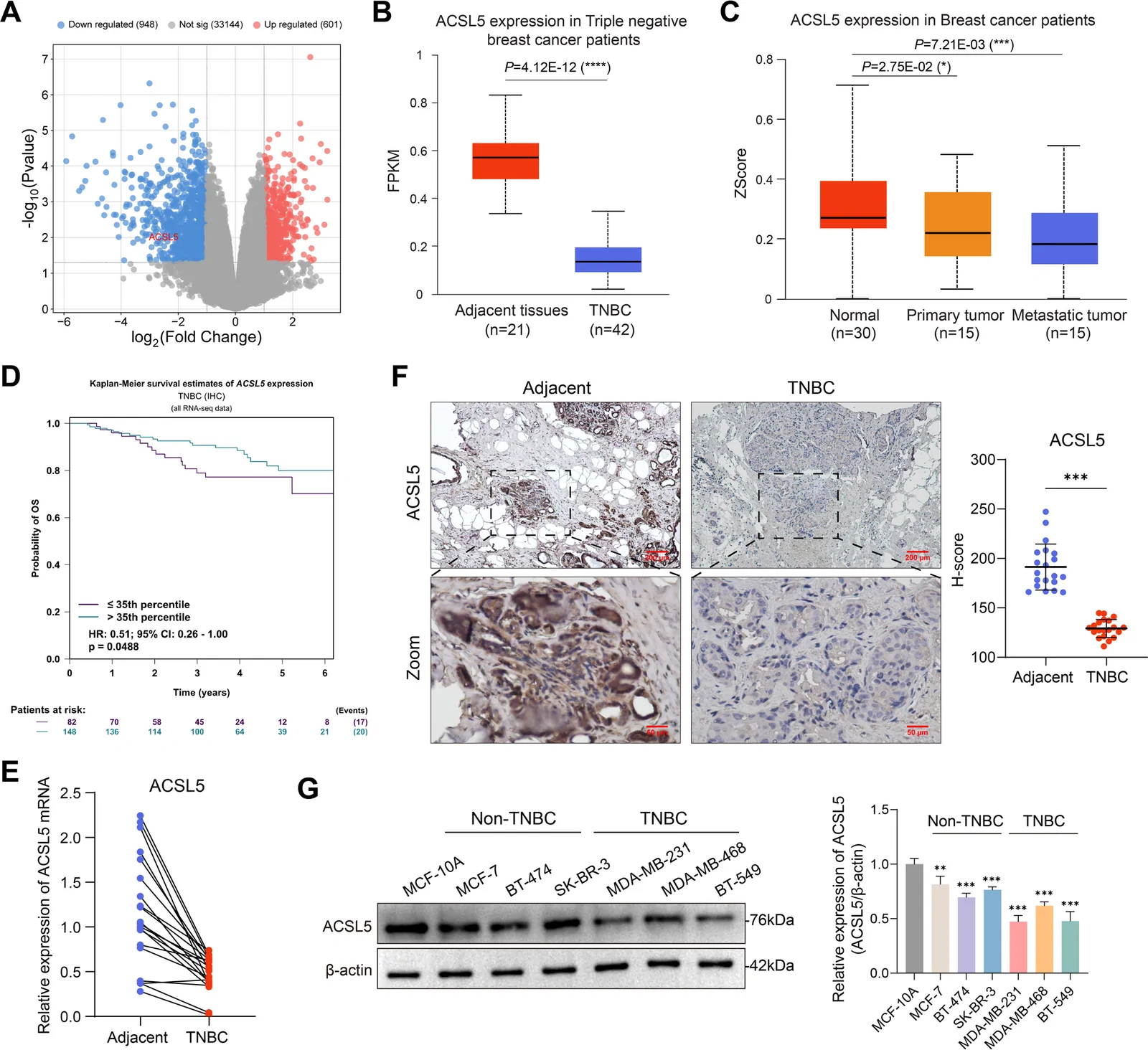
ACSL5 is downregulated in TNBC and its reduction is associated with metastatic progression and clinical relevance. **A** Based on the GEO dataset GSE113865 volcano plot of TNBC differentially expressed genes were analyzed. Blue represents downregulation, and red represents upregulation (|log2Fold Change| > 1, adj. P < 0.05). **B** The expression levels of ACSL5 mRNA in TNBC tissues (n=42) and adjacent tissues (n=21) were compared by analyzing the GEO dataset GSE58135 by the MammOnc-DB platform. **C** MammOnc-DB analyzed the GEO dataset GSE183947 to compare the expression differences of ACSL5 mRNA in primary breast cancer (n=15) and metastatic breast cancer tissues (n=15). **D** Kaplan-Meier survival analysis was performed using bc-GenExMiner v5.2 database, and the relationship between ACSL5 expression level and overall survival (OS) of TNBC patients was evaluated by log-rank test. **E** RT-qPCR validation of ACSL5 mRNA expression in 20 paired TNBC tissues and matched adjacent non-tumor tissues. **F** Immunohistochemistry (IHC) staining showed the expression of ACSL5 protein in paired adjacent non-tumor tissues and TNBC tissues, together with blinded semiquantitative H-score analysis in 20 paired clinical specimens. Representative images of 100 × (scale: 200 μm) and 400 × (scale: 50 μm) are presented. **G** Western blot detection of the expression levels of ACSL5 protein in normal breast cell lines (MCF-10A), non-TNBC breast cancer cell lines (MCF-7, BT-474 and SK-BR-3) and TNBC cell lines (MDA-MB-231, MDA-MB-468 and BT-549) and quantification analysis.Data are expressed as mean ± standard deviation (mean ± SD). Statistically significant is expressed as P < 0.05 (*), P < 0.01 (**), P < 0.001 (***) and P < 0.0001 (****)

To establish the rationale for investigating ACSL5 in TNBC, we first assessed its expression pattern across multiple independent datasets. Differential expression analysis based on GEO dataset GSE113865 showed that ACSL5 was significantly downregulated in TNBC tissues compared to non-tumor samples (Fig. 1A). This finding was further validated in an independent cohort (GSE58135), in which ACSL5 mRNA levels were significantly lower in TNBC than in adjacent tissues (*P* < 0.0001, Fig. 1B). In addition, we were surprised to find that ACSL5 expression was further reduced in metastatic breast cancer tissues compared to primary breast cancer in GSE183947 dataset (Fig. 1C), leading us to speculate that the absence of ACSL5 may be associated with metastatic progression.

Next, we evaluated the clinical relevance of ACSL5 expression and analyzed additional public datasets. Pan-cancer analysis showed that ACSL5 exhibited a generally reduced expression pattern across multiple tumor types, with a marked decrease in breast cancer (*P* < 0.0001, Supplementary Figure S1A). Further analysis based on the TCGA-BRCA cohort showed that the expression level of ACSL5 mRNA in breast cancer tissues was significantly lower than that in normal breast tissues (*P* < 0.0001, Supplementary Figure S1B). Kaplan-Meier survival analysis further showed that lower ACSL5 expression was associated with poorer overall survival in patients with TNBC (*P* < 0.05, HR = 0.51, Fig. 1D), while a similar trend was also observed in the overall BRCA cohort (*P* < 0.01, Supplementary Figure S1C). These data support the clinical significance of ACSL5 downregulation in breast cancer and TNBC. As supportive evidence, single-cell RNA-sequencing [31] analysis showed that ACSL5 expression was overall modest across multiple cell populations within the TNBC microenvironment, without indicating strong enrichment in a single lineage (Supplementary Figure S1F-G).

To further understand the expression of ACSL5 in clinical samples, we examined the expression of ACSL5 in paired TNBC tissues and adjacent non-tumor tissues. RT-qPCR analysis of 20 clinical samples showed a significant decrease in ACSL5 mRNA expression in TNBC tissues (Fig. 1E), and western blot results showed the same trend in 8 clinical samples (Supplementary Figure S1H).

Consistently, immunohistochemical staining combined with H-score showed a significant reduction in ACSL5 protein expression in TNBC tissues compared to paired neighboring tissues (Fig. 1F).

In addition, protein-level evidence from public datasets also supports reduced ACSL5 expression in breast cancer, as shown in CPTAC analysis and representative HPA staining images (Supplementary Figure S1D-E). At the cell line level, Western blot analysis showed that the expression of ACSL5 protein in TNBC cell lines was lower than that in normal breast epithelial cells and non-TNBC breast cancer cell lines (Fig. 1G). Therefore, these findings support reduced ACSL5 expression in TNBC and provide a rationale for the subsequent functional interrogation of tumor cell-intrinsic ACSL5 under adipocyte-conditioned conditions.

### ACSL5 inhibits the malignant phenotype of TNBC cells

To further clarify the functional role of ACSL5 in TNBC cells, we first measured the basal expression levels of epithelial-mesenchymal transition (EMT)-related marker proteins in a variety of breast cancer cell lines. Western blot results showed that compared with normal breast epithelial cells MCF-10 A and non-TNBC breast cancer cell lines, the expression of E-cadherin in TNBC cell lines was significantly reduced, while the expressions of N-cadherin, Snail and Vimentin were significantly increased (*P* < 0.05, Supplementary Figure S2A). Based on this, we constructed ACSL5 knockdown and overexpression models in MDA-MB-231 and BT-549 cells. RT-qPCR results showed that transfection of shRNAs with two different target sequences significantly reduced the level of ACSL5 mRNA, while the overexpression vector (OE-ACSL5) significantly up-regulated the transcription level of ACSL5 (*P* < 0.001, Supplementary Figure S2B and C). 

Western blot further verified the knockdown and overexpression efficiency of ACSL5 at the protein level (Supplementary Figures S2D and E). Because the knockdown efficiency of shACSL5-2 was better than that of shACSL5-1, shACSL5-2 was used as a knockdown group for subsequent studies.

**Fig. 2:**
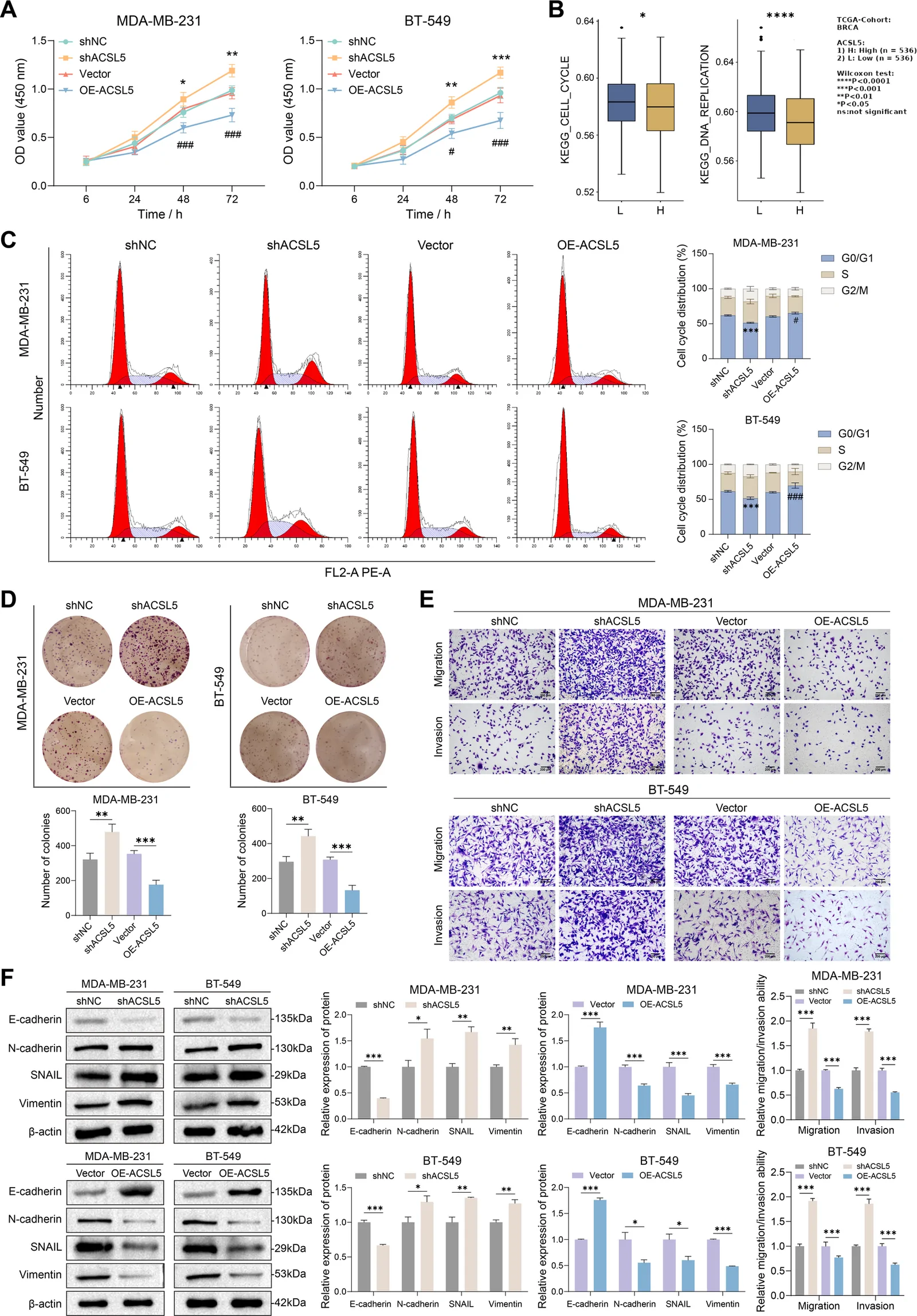
ACSL5 inhibits the proliferation and aggressive phenotype of TNBC cells. **A** CCK-8 assay to detect the effect of ACSL5 knockdown (shACSL5) or overexpression (OE-ACSL5) on changes in cell viability of MDA-MB-231 and BT-549. Compared with shNC, P < 0.05 (*), P < 0.01 (**), P < 0.001 (***) were significantly different. Compared with Vector, there was a significant difference between P < 0.05 (#) and P < 0.001 (###). **B** ssGSEA analysis of TCGA-BRCA cohorts based on the CAMOIP platform. The samples were divided into high and low expression groups according to the median expression of ACSL5, and the enrichment differences of KEGG_Cell cycle and KEGG_DNA replication pathways were compared. **C** Flow cytometry analysis of the cell cycle distribution of MDA-MB-231 and BT-549 cells after shACSL5 or OE-ACSL5. Compared with shNC, P < 0.001 (***) had a significant difference. Compared with Vector, there was a significant difference between P < 0.05 (#) and P < 0.001 (###). **D** Plate cloning assay to evaluate the effect of shACSL5 or OE-ACSL5 on the proliferation capacity of TNBC cells. **E** Transwell assay to detect the migration and invasion ability of TNBC cells after shACSL5 or OE-ACSL5. Magnification 100x (scale: 200μm). **F** Western blot was used to detect the expression changes of epithelial-mesenchymal transition (EMT)-related marker proteins (E-cadherin, N-cadherin, Snail and Vimentin) in TNBC cells after shACSL5 or OE-ACSL5. All experiments were repeated 3 times independently. Data are expressed as mean ± SD. P < 0.05 (*), P < 0.01 (**), P < 0.001 (***), P < 0.0001 (****) indicate that the results are significant

At the functional level, the CCK-8 experimental results showed that shACSL5 significantly promoted MDA-MB-231 and BT-549 cell viability compared with the control group (shNC or Vector), while OE-ACSL5 significantly inhibited cell growth (*P* < 0.05, Fig. 2A). We also verified this phenomenon from the perspective of transcriptome function and performed ssGSEA analysis based on the TCGA-BRCA cohort. The results showed that the KEGG_Cell cycle and KEGG_DNA replication pathways were significantly enriched in the ACSL5 low expression group, while the activities of these pathways were significantly reduced in the ACSL5 high expression group (*P* < 0.05, Fig. 2B), which made us realize that ACSL5 may inhibit the proliferation of TNBC cells by regulating cell cycle-related processes.

To further verify the effect of ACSL5 on cell proliferation, we first analyzed the cell cycle distribution by flow cytometry, and the results showed that shACSL5 significantly reduced the proportion of cells in G0/G1 phase, accompanied by an increase in the proportion of cells in S phase and G2/M phase. In contrast, OE-ACSL5 arrests the cell cycle at the G0/G1 phase (*P* < 0.05, Fig. 2C). Subsequent cloning experiments were conducted, and it was found that the number of clones formed in the shACSL5 group was significantly increased, while that in the OE-ACSL5 group was significantly reduced (*P* < 0.01, Fig. 2D). These results imply that ACSL5 has a negative regulatory effect on the proliferation potential of TNBC cells.

Given that increased proliferative capacity is often accompanied by increased aggressiveness, we further evaluate the effect of ACSL5 on TNBC cell migration and invasion capacity. Transwell results showed that shACSL5 significantly promoted the migration and invasion ability of MDA-MB-231 and BT-549 cells, while OE-ACSL5 significantly inhibited the above process (*P* < 0.001, Fig. 2E). The same results were shown at the molecular level, and shACSL5 was found to lead to a decrease in the expression of E-cadherin protein, while the expression of N-cadherin, Snail and Vimentin proteins was up-regulated. OE-ACSL5 showed the opposite expression trend (*P* < 0.05, Fig. 2F). These results indicate that ACSL5 suppresses TNBC cell migration and invasion, accompanied by attenuation of EMT-associated molecular changes.

### Adipocyte-derived fatty-acid cues induce SIRT6 upregulation and ACSL5 downregulation in TNBC cells

Given that ACSL5 exhibits inhibitory function in TNBC, we further focus on whether the adipocyte microenvironment is involved in ACSL5 expression imbalance. Since SIRT6 is a candidate regulator closely related to metabolic regulation and protein deacetylation, and may be involved in post-translational regulation of ACSL5, we first investigated whether adipocyte-derived stimulation synchronously affects SIRT6 and ACSL5 expression in TNBC cells. To determine whether the adipocyte microenvironment affects ACSL5 and SIRT6 expression in TNBC cells, we established an adipocyte-TNBC cell Transwell co-culture system. Mature breast adipocytes were seeded in the upper chamber, whereas MDA-MB-231 cells were cultured in the lower chamber.

**Fig. 3:**
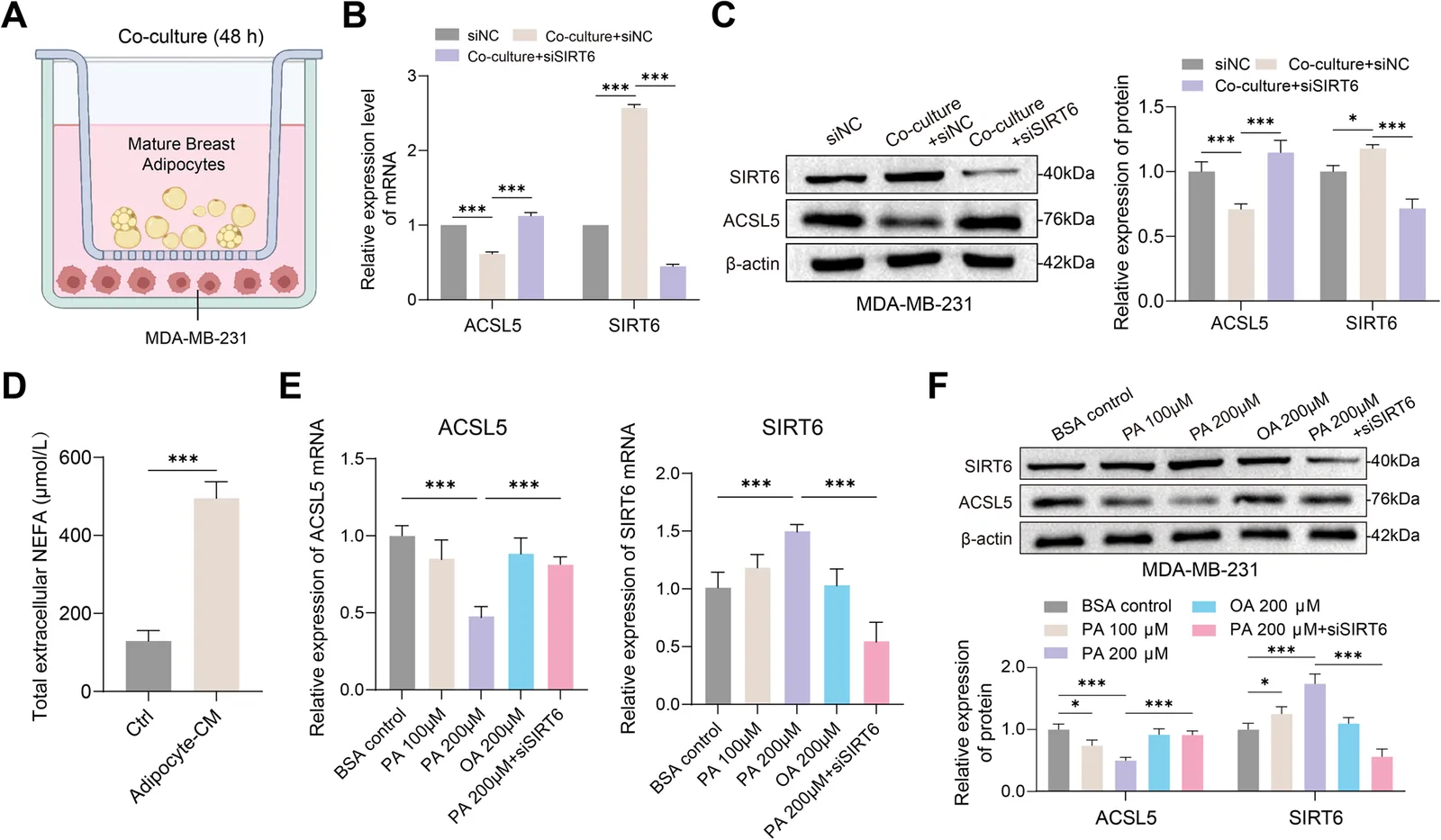
Adipocyte-derived fatty-acid cues induce SIRT6 upregulation and ACSL5 downregulation in TNBC cells. **A** Schematic diagram of Transwell co-culture system using mature breast adipocytes in the upper chamber and MDA-MB-231 cells in the lower chamber. After 48 h of co-culture, lower-chamber MDA-MB-231 cells were collected for RT-qPCR and Western blot analyses. **B** RT-qPCR was used to detect changes in ACSL5 and SIRT6 mRNA expression in MDA-MB-231 cells with or without SIRT6 knockdown under co-culture conditions. **C** Western blot analysis of changes and quantitative analysis of ACSL5 and SIRT6 protein expression in MDA-MB-231 cells with or without SIRT6 knockdown under co-culture conditions. **D** Measurement of total extracellular non-esterified fatty acid (NEFA) levels in control medium and adipocyte-conditioned medium. **E** RT-qPCR analysis of ACSL5 and SIRT6 mRNA expression in MDA-MB-231 cells with or without SIRT6 knockdown after PA or OA treatment. PA was used as a representative saturated fatty acid, whereas OA was used as a representative monounsaturated fatty acid comparator. **F** Western blot analysis and quantitative assessment of ACSL5 and SIRT6 protein expression in MDA-MB-231 cells with or without SIRT6 knockdown after PA or OA treatment. All experiments are repeated 3 times independently, and the data are expressed as mean ± SD. P < 0.05 (*), P < 0.01 (**), P < 0.001 (***) indicate statistical significance

After 48 h of co-culture, only lower-chamber MDA-MB-231 cells were collected for subsequent RT-qPCR and Western blot analyses (Fig. 3A). RT-qPCR results showed that adipocyte co-culture significantly upregulated SIRT6 mRNA expression and reduced ACSL5 mRNA expression in MDA-MB-231 cells compared with the siNC control group. Upon SIRT6 knockdown, the co-culture-associated decrease in ACSL5 mRNA expression was partially restored (Fig. 3B).

The knockdown efficiency of SIRT6 was verified in Supplementary Figure S3A and S3C.

Consistently, Western blot analysis showed that co-culture increased SIRT6 protein expression and decreased ACSL5 protein expression, whereas siSIRT6 treatment reduced SIRT6 protein expression and partially restored ACSL5 protein levels (Fig. 3C). These results indicate that adipocyte co-culture induces SIRT6 upregulation and ACSL5 downregulation in TNBC cells, and that SIRT6 is involved in co-culture-associated ACSL5 downregulation.

Next, we further examined whether adipocyte-derived fatty acid signals might be involved in these changes. The results showed that total extracellular non-esterified fatty acid (NEFA) levels were significantly increased in adipocyte-conditioned medium compared with regular culture medium, suggesting enhanced fatty acid availability in the adipocyte-conditioned environment (Fig. 3D). To further mimic fatty acid exposure, MDA-MB-231 cells were treated with palmitic acid (PA), a representative saturated fatty acid, and oleic acid (OA), a representative monounsaturated fatty acid, as a comparative control. PA treatment increased SIRT6 expression and reduced ACSL5 expression, with a more pronounced effect observed at 200 µM PA. In contrast, OA exerted a relatively weaker effect on SIRT6 and ACSL5 expression. Furthermore, SIRT6 knockdown under PA-treated conditions partially reversed PA-induced downregulation of ACSL5 at both the mRNA and protein levels (Fig. 3E and F). These results suggest that adipocyte-derived fatty acid stimulation, particularly saturated fatty acid exposure modeled by PA, may induce SIRT6 upregulation and ACSL5 downregulation in TNBC cells. It should be noted that the changes in ACSL5 mRNA and protein expression observed so far should be interpreted as SIRT6-associated expression changes under adipocyte/fatty acid conditions, rather than definitive evidence that SIRT6 directly regulates ACSL5 transcription. Therefore, in subsequent mechanistic experiments, we further examined whether SIRT6 mainly regulates ACSL5 at the post-translational level by modulating its pan-acetylation status.

### ACSL5 restoration and SIRT6 knockdown attenuate adipocyte-induced malignant phenotypes of TNBC

**Fig. 4:**
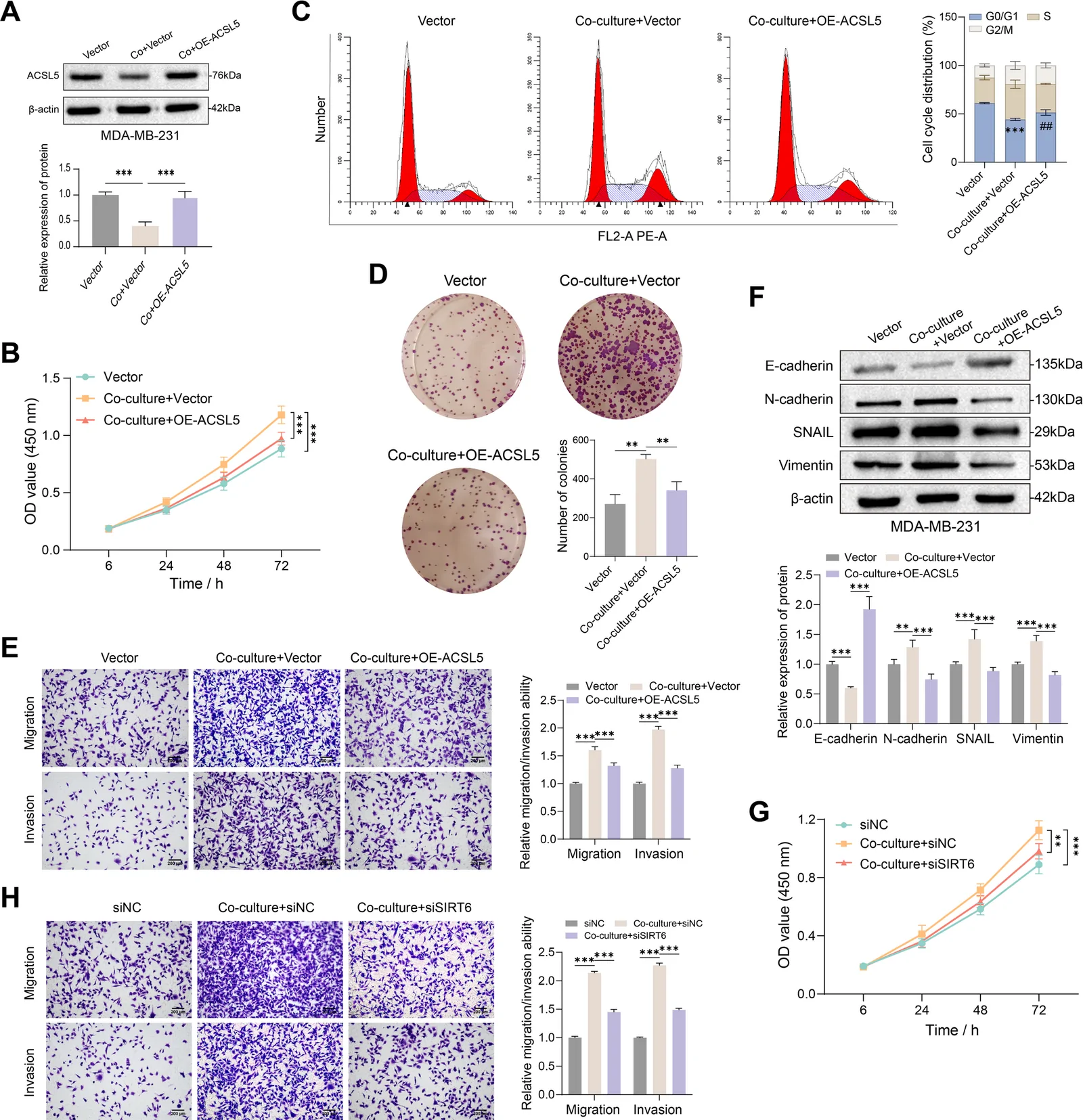
ACSL5 restoration and SIRT6 knockdown attenuate adipocyte-induced malignant phenotypes in TNBC cells. **A** Western blot analysis of ACSL5 overexpression in MDA-MB-231 cells under adipocyte co- 26 culture conditions. **B** The effects of adipocyte co-culture and ACSL5 overexpression on the proliferation of TNBC cells were detected by CCK-8 assay. **C** Flow cytometry analysis of cell cycle distribution ratios in each group. Compared with Vector, P<0.001 (***) is significant. Compared with Co-culture + Vector, P<0.01 (##) was significant. **D** Colony formation assay showing the clonogenic capacity of MDA-MB-231 cells under the indicated conditions. **E** Transwell assay to detect cell migration and invasion capacity. Magnification 100x (scale: 200μm). **F** Western blot reflects changes in the expression of EMT-related proteins. **G** CCK-8 assay showing the effect of SIRT6 knockdown on adipocyte-induced cell viability. **H** Transwell migration and invasion assays showing the effect of SIRT6 knockdown on adipocyte-induced migratory and invasive phenotypes. All experiments are repeated 3 times independently, and the data are expressed as mean ± SD. P < 0.01 (**) and P < 0.001 (***) indicate statistical significance

To further clarify whether ACSL5 is involved in the adipocyte microenvironment-induced malignant phenotype of TNBC, we performed ACSL5 overexpression in MDA-MB-231 cells under adipocyte co-culture conditions. Western blot results showed that adipocyte co-culture decreased ACSL5 protein expression in MDA-MB-231 cells, whereas ACSL5 overexpression significantly restored ACSL5 protein levels under co-culture conditions (Fig. 4A). Subsequently, we examined the effects of ACSL5 restoration on adipocyte-induced cell proliferation and colony formation. The CCK-8 results showed that the cell viability of the co-cultured + Vector group was significantly increased over time compared with the Vector group alone, and the cell proliferation curve was significantly downward after overexpression of ACSL5 under co-culture conditions, indicating that ACSL5 could partially offset the proliferation-enhancing effect induced by adipocytes (Fig. 4B).

Cell cycle flow cytometry further showed that co-culture reduced the G0/G1 population and increased the S or G2/M population, suggesting that the cell cycle advanced to the DNA replication stage. After overexpression of ACSL5 in the co-culture background, the proportion of S phase decreased and the proportion of G0/G1 phase increased, indicating that ACSL5 could inhibit adipocyte-induced cell cycle progression (Fig. 4C). The results of the clone formation experiment were consistent with the above trend, with co-culture significantly increasing the number of clones formed, while ACSL5 overexpression significantly reducing the number of clones (Fig. 4D).

Transwell experiments showed that the migration and invasion of cells in the co-culture group were significantly enhanced, while the number of migratory and invading cells was significantly reduced after ACSL5 overexpression (Fig. 4E). At the molecular level, Western blot results showed that EMT-related changes were obvious after co-culture treatment, showing that E-cadherin was down-regulated, while N-cadherin, Snail and Vimentin were up-regulated. ACSL5 overexpression reversed the above EMT protein expression pattern (Fig. 4F). The above results indicate that ACSL5 restoration can inhibit adipocyte co-culture-induced TNBC cell proliferation, colony formation, migration, invasion, and EMT-related molecular changes.

Considering that SIRT6 knockdown can partially restore ACSL5 expression under co-culture conditions, we further investigated whether SIRT6 is involved in adipocyte-induced malignant phenotypes. Results showed that adipocyte co-culture significantly enhanced MDA-MB-231 cell viability, while siSIRT6 treatment reduced co-culture-induced cell viability elevation (Fig. 4G).

SIRT6 knockdown efficiency was validated in Supplementary Figure S3. Meanwhile, Transwell results showed that SIRT6 knockdown significantly weakened the migration and invasion enhancements induced by adipocyte co-culture (Fig. 4H). Consistently, time-course analysis under co-culture and SIRT6-overexpressing conditions showed a progressive decrease in ACSL5 mRNA and protein expression, accompanied by increased SIRT6 protein expression, particularly at 48 h (Supplementary Figure S4B and S4C). In summary, these results indicate that both ACSL5 restoration and SIRT6 knockdown can partially inhibit the adipocyte microenvironment-induced TNBC malignant phenotype, suggesting that the SIRT6-ACSL5 expression axis plays a functional role in adipocyte-induced TNBC progression.

### SIRT6 interacts with ACSL5 and regulates pan-acetylation of ACSL5 at the post-translational level

**Fig. 5:**
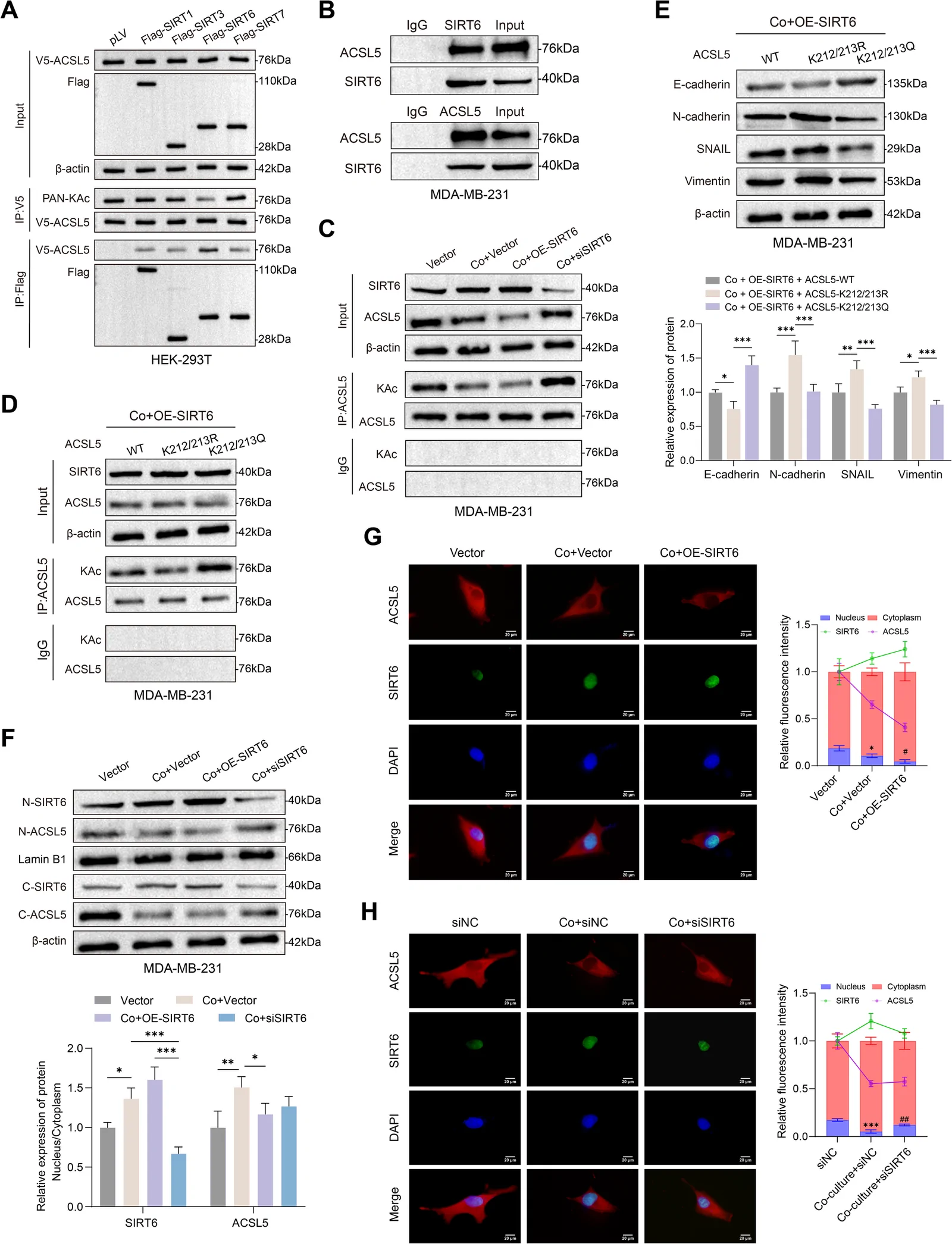
SIRT6 interacts with ACSL5 and regulates its pan-acetylation at the post-translational level. **A** Normalized screening of SIRT family members in HEK-293T cells. V5-ACSL5 was co-transfected with Flag-SIRT1, Flag-SIRT3, Flag-SIRT6, or Flag-SIRT7. The transfection amounts of each Flag-SIRT plasmid were adjusted to achieve comparable expression levels among the different SIRT proteins. After V5 immunoprecipitation (IP), ACSL5 pan-acetylation levels were detected using anti-pan-KAc antibody. **B** Endogenous co-immunoprecipitation analysis of ACSL5 and SIRT6 in MDA-MB-231 cells. IgG was used as a negative control. **C** IP of ACSL5 followed by detection of pan-KAc levels to analyze the effects of adipocyte co-culture and SIRT6 overexpression or knockdown on ACSL5 pan-acetylation. **D** IP of ACSL5 followed by detection of pan-KAc levels to analyze the effects of ACSL5-WT, ACSL5-K212/213R, and ACSL5-K212/213Q on ACSL5 pan-acetylation. K212/213R and K212/213Q are mutants of the candidate K212/213-containing lysine motif. **E** Western blot analysis of the effects of different ACSL5 mutants on the expression of EMT-related proteins (E-cadherin, N-cadherin, SNAIL, and Vimentin) under adipocyte co-culture conditions with SIRT6 overexpression. **F** Nucleo/cytoplasmic separation Western blot detection of subcellular distribution of SIRT6 and ACSL5 under different treatment conditions. Lamin B1 and β-actin serve as nuclear protein and 27 cytoplasmic protein references, respectively. **G** IF detection of subcellular localization changes in ACSL5 and SIRT6 under SIRT6 overexpression conditions. Magnification 1000x (scale: 20μm) Compared with Vector, P<0.05(*) is significant; Compared with Co-culture + Vector, P<0.05(#) was significant. **H** Immunofluorescence analysis of the subcellular localization of ACSL5 and SIRT6 under SIRT6 knockdown. Magnification 1000x (scale: 20μm) Compared with siNC, P<0.001 (***) is significant; Compared with Co-culture + siNC, P<0.01 (##) was significant. All experiments are repeated 3 times independently, and the data are expressed as mean ± SD. ns means no statistical difference; P < 0.05 (*), P < 0.01 (**), and P < 0.001 (***) indicate statistical significance

To further clarify whether SIRT6 is a candidate deacetylase regulating ACSL5, we first compared the effects of different SIRT family members on ACSL5 pan-acetylation levels. Considering that differences in the expression levels of various SIRT proteins might influence the observed deacetylation efficiency, we adjusted the transfection amounts of each Flag-SIRT plasmid to achieve comparable expression levels of SIRT1, SIRT3, SIRT6, and SIRT7. Subsequently, V5-ACSL5 was Co-transfected with different Flag-SIRT plasmids in HEK-293T cells, followed by IP with anti-V5 antibody to detect pan-KAc levels. The results showed that, under conditions of relatively normalized SIRT protein expression, SIRT6 exerted the most pronounced reducing effect on ACSL5 pan-acetylation (Fig. 5A). To further verify whether SIRT6 interacts with ACSL5, we performed endogenous co-immunoprecipitation (co-IP) assays in MDA-MB-231 cells. The results showed that ACSL5 was readily detected after immunoprecipitation with anti-SIRT6 antibody, and SIRT6 was also detected after immunoprecipitation with anti-ACSL5 antibody. No specific signal was observed in the IgG control group, indicating that ACSL5 physically interacts with SIRT6 in TNBC cells (Fig. 5B).

Subsequently, we examined whether adipocyte co-culture and SIRT6 intervention affect the pan-acetylation status of ACSL5. The results showed that, compared with the Vector group, adipocyte co-culture decreased the pan-acetylation level of ACSL5. Overexpression of SIRT6 under co-culture conditions further reduced ACSL5 pan-acetylation, whereas knockdown of SIRT6 significantly restored its pan-acetylation level (Fig. 5C). These results indicate that SIRT6 contributes to the reduction of ACSL5 pan-acetylation under adipocyte co-culture conditions, supporting the notion that SIRT6 regulates ACSL5 at the post-translational modification level. To further evaluate whether the predicted lysine motif is involved in the acetylation regulation of ACSL5, we constructed the ACSL5 wild-type (WT) construct as well as the candidate K212/213 site mutants. According to the mutation design, both adjacent lysine residues (K212 and K213) were substituted; therefore, the mutant was designated as ACSL5-K212/213R or ACSL5-K212/213Q. After immunoprecipitation of ACSL5 (IP-ACSL5), pan-KAc detection showed that, compared with wild-type ACSL5, the K212/213R and K212/213Q mutations significantly altered the pan-acetylation level of ACSL5. These results suggest that the candidate K212/213-containing lysine motif is involved in the acetylation regulation of ACSL5 (Fig. 5D).

Further examination of EMT-related proteins revealed that, under adipocyte co-culture conditions with SIRT6 overexpression, different ACSL5 mutants exerted distinct effects on the EMT molecular phenotype. Compared with ACSL5-WT, the ACSL5-K212/213R group exhibited more pronounced EMT-related changes, including decreased E-cadherin and increased N-cadherin, SNAIL, and Vimentin. In contrast, the ACSL5-K212/213Q group showed relatively attenuated EMT-associated molecular alterations (Fig. 5E). These results suggest that mutations in the K212/213-containing lysine motif not only affect the pan-acetylation status of ACSL5 but may also influence its regulatory role in EMT-related phenotypes. Considering that SIRT6 is primarily recognized as a nuclear NAD⁺-dependent deacetylase, we further examined the subcellular localization of SIRT6 and ACSL5 by nuclear/cytoplasmic fractionation followed by Western blotting. The results showed that adipocyte co-culture and SIRT6 intervention altered the nuclear/cytoplasmic distribution patterns of both SIRT6 and ACSL5. Specifically, SIRT6 overexpression enhanced the nuclear enrichment of SIRT6, whereas SIRT6 knockdown reduced its nuclear signal. The nuclear/cytoplasmic distribution of ACSL5 was also modulated by adipocyte co-culture and SIRT6 manipulation (Fig. 5F). Immunofluorescence analysis further confirmed the changes in subcellular localization of SIRT6 and ACSL5 under different treatment conditions at the morphological level (Fig. 5G and H). Collectively, these findings support that SIRT6 physically associates with ACSL5 and primarily regulates ACSL5 at the post-translational level through modulation of its pan-acetylation.

### The SIRT6-ACSL5 axis mediates adipocyte-induced lipid storage and lipid-related EMT phenotypic changes

Public dataset analysis showed that ACSL5 expression was positively correlated with the glycerolipid metabolism pathway score, suggesting a potential association between ACSL5 and lipid-related biological processes (Supplementary Figure S4A). To further determine whether the SIRT6-ACSL5 axis is associated with adipocyte-induced lipid-related phenotypic changes, we first examined intracellular lipid droplet accumulation in MDA-MB-231 cells under different treatment conditions. Oil Red O staining showed that adipocyte co-culture markedly increased intracellular lipid droplet accumulation, and this effect was further enhanced by SIRT6 overexpression.

**Fig. 6:**
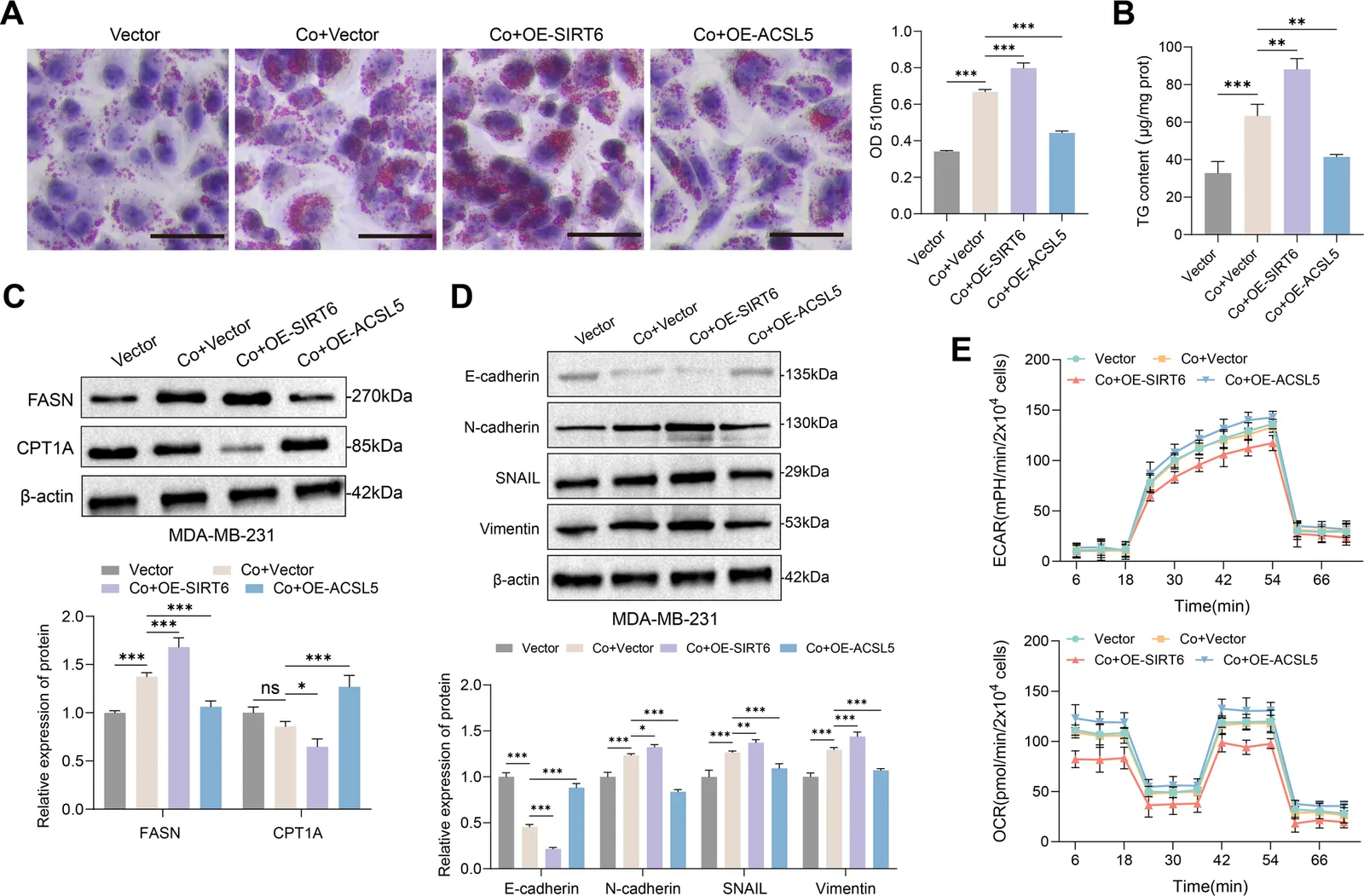
The SIRT6-ACSL5 axis is associated with altered lipid storage and lipid-related EMT phenotypes in TNBC cells. **A** Intracellular lipid droplet accumulation was detected by Oil Red O staining and semi-quantitative analysis was performed by OD 510 nm. Magnification 400x (scale: 50μm). **B** Intracellular triglyceride (TG) content under the indicated conditions. TG levels were normalized to total protein concentration and expressed as μg/mg protein. **C** Western blot detected the expression levels of proteins in key enzymes of lipid metabolism, including fatty acid synthase (FASN) and carnitine palmitoyltransferase 1A (CPT1A). **D** Western blot was used to detect the expression changes of EMT-related marker proteins. **E** The extracellular acidification rate (ECAR) and oxygen consumption rate (OCR) of TNBC cells in different treatment groups were detected by Seahorse metabolic analysis system, and the changes of glycolysis and mitochondrial oxidative metabolism levels were dynamically evaluated. All experiments are repeated 3 times independently. Data are expressed as mean ± SD. ns means no statistical significance; P < 0.05 (*), P < 0.01 (**) and P < 0.001 (***) indicate significance

Conversely, ACSL5 overexpression markedly reduced lipid droplet accumulation under co-culture conditions, and OD 510 nm quantification was consistent with the morphological observations (Fig. 6A). Consistent with Oil Red O staining, intracellular TG assay showed that adipocyte co-culture significantly increased TG content in MDA-MB-231 cells, and SIRT6 overexpression further elevated TG accumulation. In contrast, ACSL5 overexpression markedly reduced TG levels under co-culture conditions (Fig. 6B). Western blot analysis of lipid metabolism-related proteins showed that adipocyte co-culture increased the expression of the fatty acid synthesis-related protein FASN and reduced the expression of the fatty acid oxidation-related protein CPT1A. SIRT6 overexpression further enhanced this expression pattern, as reflected by higher FASN expression and lower CPT1A expression. In contrast, ACSL5 overexpression reduced FASN expression and partially restored CPT1A expression under co-culture conditions (Fig. 6C). These results suggest that the SIRT6-ACSL5 axis is associated with adipocyte-induced changes in selected lipid metabolism-related markers. Because lipid accumulation is often associated with enhanced tumor aggressiveness, we further examined EMT-associated protein expression. Western blot analysis showed that adipocyte co-culture decreased the epithelial marker E-cadherin and increased EMT-associated markers, including N-cadherin, SNAIL, and Vimentin. SIRT6 overexpression further enhanced this EMT-associated expression pattern. In contrast, ACSL5 overexpression partially reversed these EMT-associated molecular changes under co-culture conditions (Fig. 6D).

Seahorse analysis was further performed as a supportive metabolic readout. Compared with the Vector group, adipocyte co-culture altered both ECAR and OCR profiles. SIRT6 overexpression further changed these metabolic curves, whereas ACSL5 overexpression partially attenuated the co-culture-associated metabolic alterations (Fig. 6E). These Seahorse data were interpreted as supportive evidence of treatment-associated metabolic changes and were considered together with lipid droplet staining, TG quantification, and lipid metabolism-related protein analysis. Overall, adipocyte co-culture and SIRT6 overexpression were associated with increased lipid storage, altered lipid metabolism-related marker expression, and enhanced EMT-associated phenotypes in TNBC cells, whereas ACSL5 restoration partially attenuated these changes. These findings provide functional support that the SIRT6-ACSL5 axis is linked to adipocyte-induced lipid storage and lipid-related malignant phenotypes, thereby supporting subsequent in vivo validation in the orthotopic mammary fat pad model.

**ACSL5 suppresses TNBC tumor growth**,** spontaneous lung metastasis**,** and lipid-associated phenotypes in an orthotopic mammary fat pad model**.

**Fig. 7:**
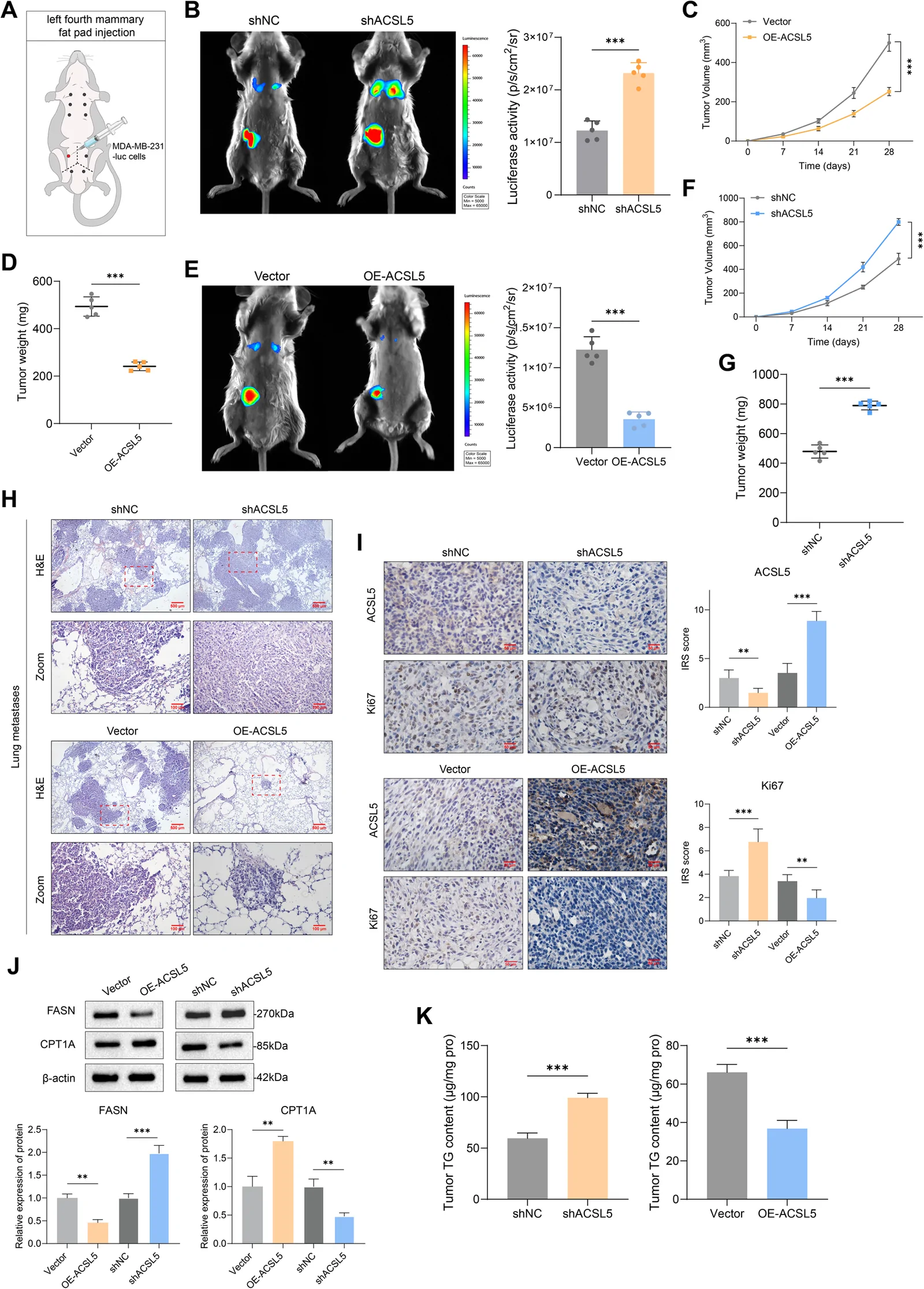
ACSL5 suppresses TNBC progression and lipid-associated phenotypes in an orthotopic mammary fat pad model. **A** Schematic illustration of orthotopic injection of MDA-MB-231-luc cells into the left fourth mammary fat pad of female NOD/SCID mice. **B** Representative endpoint whole-body bioluminescence images and quantification of luciferase activity in the shNC and shACSL5 groups. Each group of 5 mice. **C** Tumor growth curves of orthotopic tumors in the Vector and OE-ACSL5 groups. **D** Endpoint tumor weight in the Vector and OE-ACSL5 groups. **E** Representative endpoint whole-body bioluminescence images and quantification of luciferase 28 activity in the Vector and OE-ACSL5 groups. Each group of 5 mice. **F** Tumor growth curves of orthotopic tumors in the shNC and shACSL5 groups. **G** Endpoint tumor weight in the shNC and shACSL5 groups. **H** H&E staining of lung tissues showing spontaneous metastatic involvement from orthotopic mammary tumors. Low-magnification and enlarged images are shown. **I** IHC staining and IRS quantification of ACSL5 and Ki67 expression in orthotopic tumor tissues. **J** Western blot analysis and quantification of the lipid metabolism-related proteins FASN and CPT1A in orthotopic tumor tissues. β-actin was used as the loading control. **K** TG content in orthotopic tumor tissues. TG levels were normalized to total protein concentration and expressed as μg/mg protein. Experimental data are expressed as mean ± SD. Statistical analysis is indicated as P < 0.05 (*), P < 0.01 (**) and P < 0.001 (***)

To further validate the role of ACSL5 in a more physiologically relevant mammary adipose microenvironment, we established an orthotopic mammary fat pad xenograft model by injecting MDA-MB-231-luc cells into the left fourth mammary fat pad of female NOD/SCID mice (Fig. 7A). Endpoint whole-body bioluminescence imaging showed that ACSL5 knockdown markedly increased the in vivo luciferase signal compared with the shNC group, indicating an increased orthotopic tumor burden (Fig. 7B). Conversely, ACSL5 overexpression reduced the bioluminescent signal compared with the Vector group, suggesting decreased in vivo tumor burden (Fig. 7E). Of note, whole-body bioluminescence imaging was used primarily to reflect overall orthotopic tumor burden, whereas lung metastatic involvement was further evaluated by H&E staining of lung tissues. Consistently, tumor growth curves showed that ACSL5 overexpression suppressed orthotopic tumor growth, whereas ACSL5 knockdown promoted tumor growth (Fig. 7C and F). Endpoint tumor weight analysis further confirmed that OE-ACSL5 tumors were lighter than Vector tumors, while shACSL5 tumors were heavier than shNC tumors (Fig. 7D and G).

These results indicate that ACSL5 suppresses TNBC tumor growth in the orthotopic mammary fat pad microenvironment.

We next evaluated spontaneous lung metastatic dissemination from orthotopic tumors. H&E staining of lung tissues showed increased metastatic lesions in the shACSL5 group compared with the shNC group, whereas ACSL5 overexpression reduced lung metastatic burden compared with the Vector group (Fig. 7H). IHC analysis of orthotopic tumor tissues further confirmed reduced ACSL5 staining and increased Ki67 staining in shACSL5 tumors. In contrast, OE-ACSL5 tumors showed increased ACSL5 expression and reduced Ki67 expression (Fig. 7I). These findings support the inhibitory role of ACSL5 in tumor cell proliferation and lung metastatic involvement in vivo. Because our in vitro data suggested that ACSL5 is associated with lipid storage and lipid-related phenotypes, we further examined lipid metabolism-related markers in orthotopic tumor tissues. Western blot analysis showed that ACSL5 knockdown increased FASN expression and decreased CPT1A expression, whereas ACSL5 overexpression reduced FASN expression and partially restored CPT1A levels (Fig. 7J). In addition, tumor TG content was increased in the shACSL5 group and reduced in the OE-ACSL5 group (Fig. 7K). These findings were consistent with the in vitro Oil Red O staining, TG assay, and FASN/CPT1A results, suggesting that ACSL5 suppresses lipid storage and lipid-related marker changes in TNBC tumors within the mammary fat pad microenvironment.

Together, these orthotopic mammary fat pad data demonstrate that ACSL5 suppresses TNBC tumor growth, lung metastatic involvement, proliferative activity, and lipid-associated phenotypes in vivo. Complementary subcutaneous xenograft assays showed consistent trends, with ACSL5 knockdown promoting tumor growth and ACSL5 overexpression suppressing tumor growth (Supplementary Figure S5). Collectively, these findings support the inhibitory role of ACSL5 in TNBC progression in vivo and further link ACSL5 restoration to reduced lipid-associated phenotypes in the orthotopic mammary fat pad microenvironment.

**Fig. 8:**
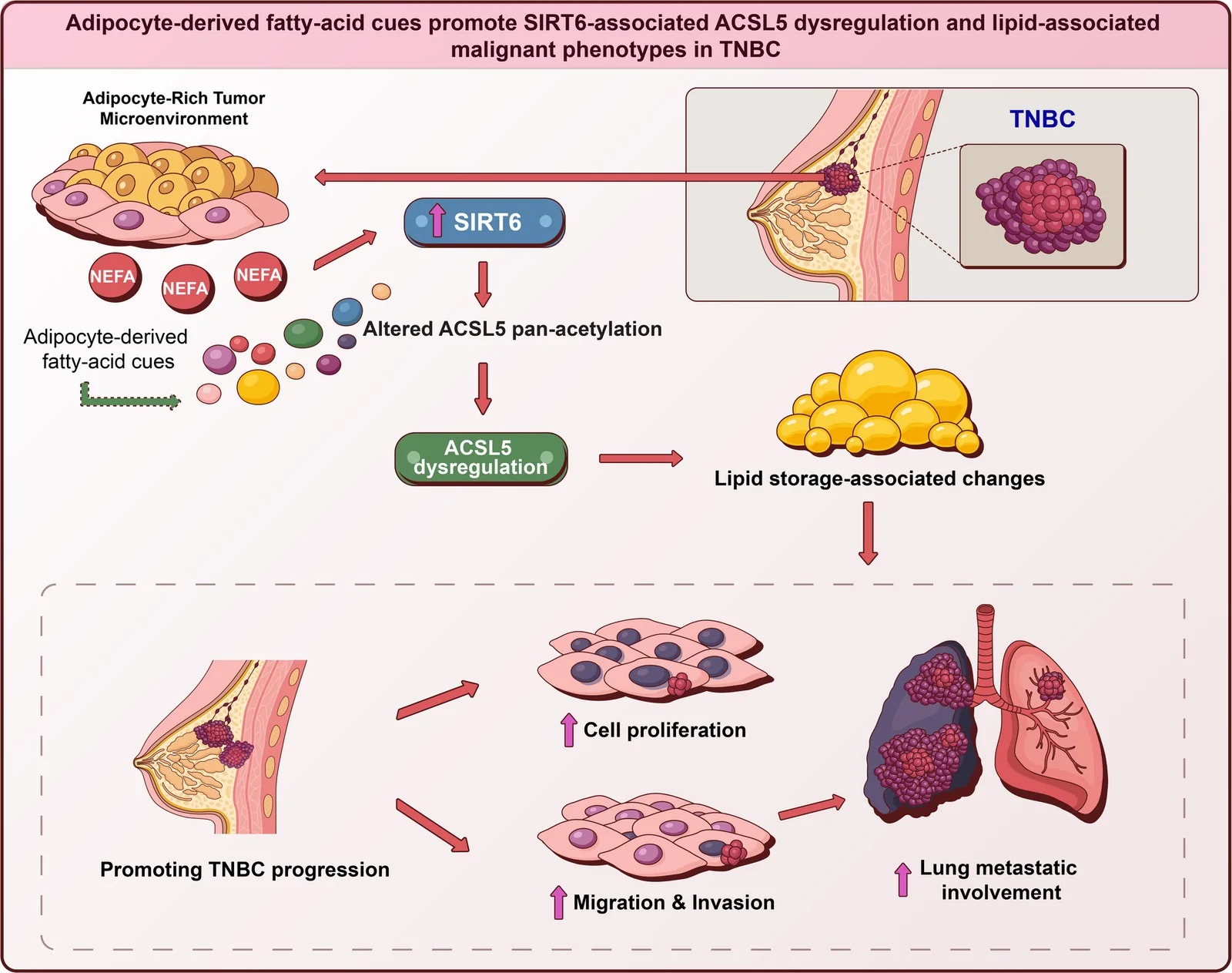
Adipocyte-rich microenvironment promotes TNBC progression through the SIRT6-ACSL5 axis and lipid-associated phenotypic changes. In the adipocyte-rich mammary microenvironment, adipocyte-derived fatty-acid cues induce SIRT6 upregulation and ACSL5 downregulation in TNBC cells. SIRT6 interacts with ACSL5 and regulates ACSL5 pan-acetylation, contributing to ACSL5 dysregulation. This process is associated with lipid storage-associated changes and enhanced malignant phenotypes, including increased proliferation, migration, invasion, and lung metastatic involvement

Based on the above in vitro and *in viv*o findings, we proposed a working model to summarize the potential role of the adipocyte-SIRT6-ACSL5 axis in TNBC progression (Fig. 8). In the adipocyte-rich mammary microenvironment, adipocyte-derived fatty-acid cues induce SIRT6 upregulation and ACSL5 downregulation in TNBC cells. SIRT6 interacts with ACSL5 and is involved in regulating ACSL5 pan-acetylation, thereby contributing to ACSL5 functional dysregulation. This process is associated with lipid storage-associated changes and enhanced malignant phenotypes, including increased proliferation, migration, invasion, and lung metastatic involvement. Conversely, ACSL5 restoration reduces lipid storage and attenuates lipid-associated malignant phenotypes. Together, this model suggests that the adipocyte-rich microenvironment may promote TNBC progression partly through SIRT6-associated ACSL5 dysregulation and lipid storage-associated phenotypic changes.

## Discussion

In recent years, the role of the tumor microenvironment in tumor progression has attracted increasing attention, particularly the abundant adipocytes in breast tissue, which may regulate tumor lipid storage-associated phenotypes and invasive progression [32]. The role of the tumor microenvironment in tumor progression has attracted increasing attention, particularly the abundant adipocytes in breast tissue, which may regulate tumor lipid storage-associated phenotypes and invasive progression [33]. However, there is still a lack of systematic research on how the adipocyte microenvironment participates in the regulation of TNBC malignant behavior [34, 35]. In this study, multi-database analysis combined with in vitro and in vivo experiments systematically revealed the potential inhibitory effect of ACSL5 in the progression of TNBC, and further explored the regulatory mode of adipocyte microenvironment.

Lipid metabolism-related enzymes often show situation-dependent effects in different tumor types [36], and there are also functional differentiation phenomena in ACSL family members [35, 37]. 

In this study, ACSL5 was observed to be closer to the inhibitory role in the TNBC system, which was consistent across multiple layers of evidence, including transcriptional levels, protein levels, cell function experiments, and in vivo tumorigenesis and metastasis results. Pathway enrichment analysis showed that ACSL5 low expression samples were activated with cell cycle and DNA replication pathway, and ACSL5 knockdown promoted colony formation and cell cycle advancement in functional experiments. Combined with the changes in EMT markers, it suggests that ACSL5 may be involved in the coordinated regulation between metabolic state and invasion phenotype.

The breast adipocyte microenvironment is another central factor of this study [7, 38]. In the co-culture model, adipocytes could stably enhance the migration and invasion ability of TNBC cells, and the downregulation of ACSL5 and the increase of lipid metabolism activation indexes showed that adipocyte-derived cues may influence lipid storage-associated phenotypes and malignant behavior of tumor cells [39, 40]. In this context, changes in the NAD⁺-dependent deacetylase SIRT6 are of particular interest. In this study, SIRT6 was upregulated under adipocyte co-culture or fatty-acid stimulation and was found to interact with ACSL5. Mechanistically, SIRT6 overexpression reduced ACSL5 pan-acetylation, whereas SIRT6 knockdown restored ACSL5 pan-acetylation under co-culture conditions. Mutagenesis of the candidate K212/213-containing lysine motif further suggested that this region may contribute to ACSL5 acetylation-associated functional regulation. More importantly, ACSL5 restoration partially attenuated the malignant phenotypes induced by adipocyte co-culture or SIRT6 upregulation, supporting a functional link among adipocyte-derived cues, SIRT6-associated ACSL5 pan-acetylation changes, and TNBC malignant phenotypes.

The metabolic phenotypic results provide a functional landing point for the above regulatory axis. Adipocyte co-culture and SIRT6 overexpression were accompanied by increased lipid droplet accumulation, elevated TG storage, increased FASN expression, reduced CPT1A expression, and altered metabolic readouts, whereas ACSL5 restoration partially reversed these lipid storage-associated changes and attenuated EMT-associated protein expression changes. Lipid storage-associated phenotypic changes are thought to be closely related to migratory invasion capacity [41], and the results of this study support a synergistic relationship between metabolic state changes and invasion phenotypes. ACSL5 is a key step in fatty acid activation, and its functional state changes may affect lipid utilization pathways and intracellular lipid signaling environment, thereby indirectly affecting the initiation intensity of invasion-related programs.

In vivo experimental results further supported these findings. To better reflect the adipocyte-rich mammary microenvironment, we established an orthotopic mammary fat pad model using MDA-MB-231-luc cells in female NOD/SCID mice. In this model, ACSL5 knockdown increased bioluminescent tumor burden, accelerated tumor growth, enhanced Ki67 expression, and increased lung metastatic involvement, whereas ACSL5 overexpression produced the opposite effects. Tumor TG content and FASN/CPT1A expression changes were also consistent with the in vitro lipid-related findings. In addition, the original subcutaneous xenograft assays were retained as complementary in vivo evidence and showed consistent trends, with ACSL5 knockdown promoting tumor growth and ACSL5 overexpression suppressing tumor growth. This study suggests a potential regulatory relationship between adipocyte-derived fatty-acid cues, SIRT6-associated ACSL5 dysregulation, lipid storage-associated phenotypic changes, and TNBC malignant progression, providing a new perspective for understanding adipocyte-driven TNBC progression.

These findings suggest that ACSL5 may represent a lipid-related regulatory node linking the adipocyte-rich microenvironment to TNBC progression and may provide a potential direction for attenuating adipocyte-driven malignant phenotypes. Several limitations should be noted. The exact ACSL5 acetylation residue was not identified by LC-MS/MS, and direct transcriptional regulation of ACSL5 by SIRT6 was not established. In addition, PA and OA were used as representative fatty-acid stimuli and may not fully reflect the complexity of the adipocyte-derived lipid environment. In summary, our findings suggest that adipocyte-derived fatty-acid cues promote TNBC progression partly through SIRT6-associated ACSL5 dysregulation and lipid storage-associated phenotypic changes, highlighting ACSL5 as a potential regulatory node in adipocyte-driven TNBC progression.

## Conclusion

In conclusion, our study demonstrates that ACSL5 functions as a suppressive lipid metabolism-related regulator in TNBC. Adipocyte-derived fatty-acid cues induce SIRT6 upregulation and ACSL5 downregulation, while SIRT6 interacts with ACSL5 and regulates ACSL5 pan-acetylation. ACSL5 restoration attenuates lipid droplet accumulation, TG storage, EMT-associated malignant phenotypes, tumor growth, and lung metastatic involvement. These findings suggest that the adipocyte-rich mammary microenvironment may promote TNBC progression partly through SIRT6-associated ACSL5 dysregulation and lipid storage-associated phenotypic changes, highlighting ACSL5 as a potential regulatory node for adipocyte-driven TNBC progression. 

## Supplementary Information

Supplementary Material 1: Figure S1. Supportive public-dataset and single-cell evidence for reduced ACSL5 expression in breast cancer and TNBC. (A) Based on the TCGA database, the TCGAplot package in R language was used to analyze pan-cancer of 33 cancers, and the expression levels of ACSL5 mRNA in tumor tissues and normal tissues were compared. (B) Analysis of the expression of ACSL5 mRNA in breast cancer tissue (n = 1099) versus normal breast tissue (n = 292) in the TCGA-BRCA cohort by the GEPIA 3.0 platform. (C) Kaplan-Meier survival analysis evaluated the relationship between ACSL5 expression levels and overall survival (OS) in breast cancer patients. The patients were divided into high and low expression groups according to the median expression of ACSL5, and the difference in survival was evaluated by log-rank test. (D) Analysis of the expression levels of ACSL5 protein in breast cancer tissues and normal breast tissues based on the CPTAC database. (E) The HPA database shows representative images of ACSL5 protein immunohistochemical staining of normal breast tissues and breast cancer tissues. (F) Cell clustering analysis of TNBC samples based on single-cell RNA sequencing data from the MammOnc-DB platform, UMAP plot showing the distribution of different cell types (left) and violin plot showing the expression of ACSL5 in different cell types (right). (G) UMAP plots show the expression and distribution of ACSL5 in different cell populations. (H) Western blot was used to detect the expression of ACSL5 protein in 8 patients paired with TNBC tissue and quantitative analysis. Statistically significant is expressed as P < 0.05 (*), P < 0.01 (**), P < 0.001 (***) and P < 0.0001 (****).

Supplementary Material 2: Figure S2. Basal EMT marker expression in breast cell lines and validation of ACSL5 knockdown and overexpression efficiency in TNBC cells. (A) Western blot detected the expression levels of EMT-related marker proteins (E-cadherin, N-cadherin, Snail, and Vimentin) in normal breast epithelial cell lines, non-TNBC breast cancer cell lines, and TNBC cell lines. RT-qPCR was used to detect the expression changes of ACSL5 mRNA after ACSL5 knockdown (B) or overexpression (C) in MDA-MB-231 and BT-549 cells. The expression levels of ACSL5 protein in MDA-MB-231 and BT-549 cells after ACSL5 knockdown (D) or overexpression (E) were detected by Western blot. All experiments are performed 3 independent replicates. Data are expressed as mean ± SD. ns means that the difference is not statistically significant; P< 0.05 (*), P< 0.01 (**), and P< 0.001 (***) indicate significant differences.

Supplementary Material 3: Figure S3. SIRT6 knockdown and overexpression efficiency in MDA-MB-231 cells. RT-qPCR was used to detect the expression level of mRNA after SIRT6 interference (A) or overexpression (B) in MDA-MB-231 cells. Western blot detected the protein expression level of SIRT6 after interference (C) or overexpression (D) and quantitative analysis. All experiments are performed 3 independent replicates. Data are presented as mean ± SD. ns means no significance; P < 0.01 (**) and P < 0.001 (***) indicate statistical significance.

Supplementary Material 4: Figure S4. Public-dataset association of ACSL5 with lipid-related pathways and time-course analysis of ACSL5 expression under SIRT6-overexpressing co-culture conditions. (A) Correlation analysis showing the association between ACSL5 expression and the glycerolipid metabolism pathway score. (B) RT-qPCR analysis of SIRT6 and ACSL5 mRNA expression in MDA-MB-231 cells under adipocyte co-culture and SIRT6-overexpressing conditions at the indicated time points. (C) Western blot analysis and quantification of SIRT6 and ACSL5 protein expression under adipocyte co-culture and SIRT6-overexpressing conditions at the indicated time points. β-actin was used as the loading control. Data are presented as mean ± SD. P < 0.05 (*), P < 0.01 (**); ns, not significant.

Supplementary Material 5: Figure S5. Complementary subcutaneous xenograft assays supporting the in vivo role of ACSL5. (A) Representative images of mice and excised tumors from the shNC and shACSL5 subcutaneous xenograft groups. (B) Tumor growth curve, endpoint tumor volume, and tumor weight in the shNC and shACSL5 groups. (C) Representative images of mice and excised tumors from the Vector and OE-ACSL5 subcutaneous xenograft groups. (D) Tumor growth curve, endpoint tumor volume, and tumor weight in the Vector and OE-ACSL5 groups. Six mice per group. Data are presented as mean ± SD. P < 0.05 (*), P < 0.01 (**).

## Data Availability

The data used and analyzed in this study may be obtained from the corresponding author upon reasonable request.

## Abbreviations

ACSL5: Acyl-CoA synthetase long chain family member 5
Co-IP: Co-immunoprecipitation
ECAR: Extracellular acidification rate
EMT: Epithelial-mesenchymal transition
IF: Immunofluorescence
IHC: Immunohistochemistry
NEFA: Non-esterified fatty acid
OA: Oleic acid
OCR: Oxygen consumption rate
PA: Palmitic acid
RT-qPCR: Reverse transcription quantitative PCR
SIRT6: Sirtuin 6
TG: Triglyceride
TNBC: Triple-negative breast cancer
